# Myocardial dysfunction caused by MyBPC3 P459fs mutation in hypertrophic cardiomyopathy: evidence from multi-omics approaches and super-resolution imaging

**DOI:** 10.3389/fcvm.2025.1529921

**Published:** 2025-02-27

**Authors:** Yupeng Wu, Yuzhu Zhang, Qirui Zheng, Qiyuan Wang, Xingyu Fang, Zaihan Zhu, Jing Lu, Dandan Sun

**Affiliations:** ^1^ Department of Ultrasound, The People’s Hospital of China Medical University, The People’s Hospital of Liaoning Province, Shenyang, China; ^2^ Department of Neurosurgery, The People’s Hospital of China Medical University, The People’s Hospital of Liaoning Province, Shenyang, China; ^3^ Shenyang Clinical Medical Research Center for Ultrasound, The People’s Hospital of China Medical University, The People’s Hospital of Liaoning Province, Shenyang, China; ^4^ Beijing Advanced Innovation Center for Food Nutrition and Human Health, Beijing Key Laboratory of Flavor Chemistry, School of Food and Health, Beijing Technology and Business University, Beijing, China

**Keywords:** hypertrophic cardiomyopathy, p459fs mutation, cardiac dysfunction, myocardial disarray, pathway

## Abstract

**Introduction:**

Mutations in the sarcomere protein, particularly in cardiac myosin binding protein C gene (*MyBPC3*), were the most frequent genetic cause of hypertrophic cardiomyopathy (HCM). The pathogenic MyBPC3 P459fs mutation has been reported in HCM patients. However, there was limited knowledge of the structure–function relationships and potential pathways in clinical HCM with MyBPC3 P459fs mutation.

**Methods:**

We used multi-omics approaches and super-resolution imaging to explore the effects of MyBPC3 P459fs mutation on humans and cells. HCM patients carrying MyBPC3 P459fs mutation (MyBPC3-P459fs HCMs) and healthy controls (HCs) were evaluated for myocardial function using both conventional and advanced echocardiography. In parallel, H9C2 myocardial cells infected with either MyBPC3 P459fs mutation (P459fs cells) or its wild type (WT cells) were investigated for myocardial fiber formation and the potential pathways behind this using super-resolution imaging and metabolomics and proteomics.

**Results:**

First, conventional and advanced echocardiography showed that MyBPC3-P459fs HCMs exhibited left ventricular diastolic and systolic dysfunction. Subsequently, super-resolution imaging indicated that P459fs cells formed fewer and shorter myocardial fibers in the cytoplasm compared to WT cells. Moreover, our metabolomic and proteomic data suggested several key components of mitochondrial membrane integrity, myocardial remodeling, myocardial energy metabolism, oxidative stress, inflammation, and actin binding capacity were significantly altered in response to P459fs mutation.

**Conclusions:**

This investigation indicated myocardial dysfunction and myocardial fiber disarray in clinical HCMs with MyBPC3 P459fs mutation and added potential pathways underlying this. These findings provided a link between the observed structural and functional disorders in MyBPC3 P459fs mutation and its onset of HCM pathogenesis and might have a significant translational contribution to effective treatment in HCM patients with MyBPC3 P459fs mutation.

## 1 Introduction

Hypertrophic cardiomyopathy (HCM) is a genetic cardiac disease affecting approximately 1 in 200–500 individuals (1). It is characterized by ventricular hypertrophy, myocyte disarray, and interstitial fibrosis (2). HCM patients experience sudden cardiac death, with an annual mortality rate of approximately 1% (3). The most frequent genetic cause of HCM worldwide is the cardiac myosin binding protein C gene (*MyBPC3*) mutation, accounting for approximately 50% of all cases (4). Cardiac myosin binding protein C (cMyBP-C) encoded by the *MyBPC3* gene is a sarcomeric thick filament protein that localizes in regular intervals at the C zone of the sarcomere to regulate the structure and function of myocardial fibers (5). The common *MyBPC3* mutations typically include truncation, frameshift, and splice variants (6). These mutations, in turn, cause haploinsufficiency, leading to HCM (7). To date, over 200 mutations in *MyBPC3* alone have been described, with variable cardiac functions ranging from nearly hypercontraction to extremely life-threatening hypocontraction (8, 9). Approximately 60% of *MyBPC3* mutations are frameshift mutations, which destroy the reading frames, produce stop codons, generate truncated carboxyl terminals, and result in deficient binding sites of myosin-binding protein (10).

A wide range of studies have attempted to elucidate how *MyBPC3* mutations change the cardiac function of HCM patients. For example, Toepfer et al. found that *MyBPC3* truncating mutations could cause increases in actin sliding velocity, ATPase activity, and force generation (8). These results suggested a gain of function or hypercontraction in HCM patients with *MyBPC3* truncating mutations. Conversely, the study by Barefield et al. on mice carrying *MyBPC3* truncating mutation suggested decreased contraction, as supported by decreases in power output, maximum shortening velocity, and single myosin force production (11). Because of these controversial findings, whether *MyBPC3* mutations result in a gain or a loss of myocardial function remains elusive. Moreover, Kuster et al. found that transgenic mice carrying *MyBPC3* mutation in the C10 domain exhibit hypercontraction hearts with higher ejection fraction (EF) when compared to non-transgenic mice (12). Blagova et al. reported a patient with HCM with MyBPC3 p.2711_2737del mutation with decreased contraction of 24% EF (13). These findings raised concerns as to what the exact cardiac function pattern would be in HCM with different *MyBPC3* mutations. A comprehensive evaluation of the phenotype caused by a specific *MyBPC3* mutation and its underlying pathways is needed to delay the progression of HCM and for greater potential to substantially impact prognosis and outcomes.

Yang et al. first reported the MyBPC3 P459fs mutation in a large family pedigree. Four familial members carrying the MyBPC3 P459fs mutation were diagnosed with HCM on echocardiography (14). Recently, Qiu et al. also identified MyBPC3 P459fs in four sporadic individuals who developed HCM in middle age (15). In agreement with the above findings, we identified cytosine nucleotide deletion (c. 1377delC) in exon 15 of *MyBPC3*, leading to the identical frameshift mutation in *MyBPC3* (p. P459fs). Recently, Wang et al. established one human induced pluripotent stem cell line (iPSC) carrying a heterozygous P459fs mutation in *MyBPC3*, which had the capacity to differentiate into derivatives of all three germ layers (16). This cell line exhibited a variety of deleterious phenotypes in response to angiotensin II, including reduced *MyBPC3* expression, hypertrophy, arrhythmia, and elevated diastolic intracellular Ca^2+^ (15). The above studies provided little evidence of a decrease in left ventricular (LV) EF, and there remains a lack of comprehensive data on early cardiac function changes. Further investigation is needed to elucidate the pathogenic role of MyBPC3 P459fs mutation in HCM patients.

MyBPC3 P459fs mutations were identified in five unrelated HCM patients using whole-exome sequencing (WES) and panel sequencing. Echocardiography exhibited severe interventricular septum hypertrophy without left ventricular outflow tract obstruction and normal LVEF. Here, we used conventional and advanced echocardiography to identify the alterations of LV diastolic and systolic function in HCM patients with MyBPC3 P459fs mutation. Subsequently, to address how MyBPC3 P459fs mutation led to the aforementioned phenotypes, we infected H9C2, a myocardial cell line, with either MyBPC3 P459fs mutation or its wild-type (WT) counterpart. Super-resolution imaging was performed to assess the subcellular distribution of MyBPC3 P459fs mutant or its WT counterpart in cells. Intriguingly, MyBPC3 P459fs mutation led to the corresponding alterations of myocardial fibers in the cytoplasm. To explore the potential pathways underlined, we examined the metabolomic and proteomic profiles. As expected, several myocardial pathways were significantly altered. This work herein highlighted the potential treatment targeting of MyBPC3 P459fs mutation in HCM patients, especially those with cardiac dysfunction.

## 2 Materials and methods

### 2.1 Participants

Participants included five HCM patients with MyBPC3 P459fs mutation (MyBPC3-P459fs HCMs) and 10 healthy controls (HCs). MyBPC3-P459fs HCMs and HCs were recruited from the Department of Cardiac Function at the People's Hospital of Liaoning Province. This study was approved by the Ethics Committee of the People's Hospital of Liaoning Province (2022KS007). The study was conducted in line with the principles of the 1975 Declaration of Helsinki. Written informed consent was obtained from each participant. MyBPC3-P459fs HCMs were diagnosed as follows. First, maximal wall thickness (MWT) was ≥15 mm involving ≥1 segments of LV or ≥13 mm in the first-degree relatives of HCM patients, which could not be explained by loading conditions. Second, DNA sequencing revealed a frameshift mutation, P459fs, deleting one nucleotide (cytosine) in exon 15 of *MyBPC3*. HCs did not have HCM and any first-degree relatives with a history of HCM. There was no MyBPC3 P459fs mutation in DNA sequencing. Individuals were excluded if they had the following conditions: ischemic heart disease, heart valve disease, cardiac arrhythmia, severe kidney or liver disease, and malignant disease. Our study examined male and female individuals, and similar findings were reported for both sexes.

Rat H9C2 myocardial cell lines were purchased from the American Type Culture Collection (ATCC, Manassas, USA). Stably transfected H9C2 cell lines were grouped into cells with MyBPC3 P459fs mutation (P459fs cells) and cells with MyBPC3 WT.

### 2.2 Study design

Study 1 assessed the changes of myocardial function in MyBPC3-P459fs HCMs and HCs using conventional echocardiography and two-dimensional speckling tracking echocardiography. Study 2 investigated the effects of MyBPC3 P459fs mutation in P459fs cells and WT cells using super-resolution imaging. Study 3 explored the potential pathways of MyBPC3 P459fs mutation in myocardial functional changes using metabolomic and proteomic profiles in P459fs cells and WT cells.

### 2.3 Mutation screening

Genomic DNA was extracted from the whole blood sample of each participant. First, WES was conducted to identify the MyBPC3 P459fs mutation in the first MyBPC3-P459fs HCM. Exons and flanking intronic regions were captured by the SureSelect Human All Exon System (Agilent Technologies, USA) and then sequenced on the HiSeq 2,500 Sequencing System (Illumina, USA) in PE150 format with an average sequencing depth of 25×. Second, targeted region sequencing was performed to screen the MyBPC3 P459fs mutation in the other four MyBPC3-P459fs HCMs and 10 HCs. Multiplex PCR primers were designed to capture all 35 exons and flanking sites of *MyBPC3* using the MultipSeq AImumiCap Panel (iGene Tech, China). Amplified enriched DNA was sequenced with the HiSeq Xten sequencing system (Illumina, USA).

**Figure 1:**
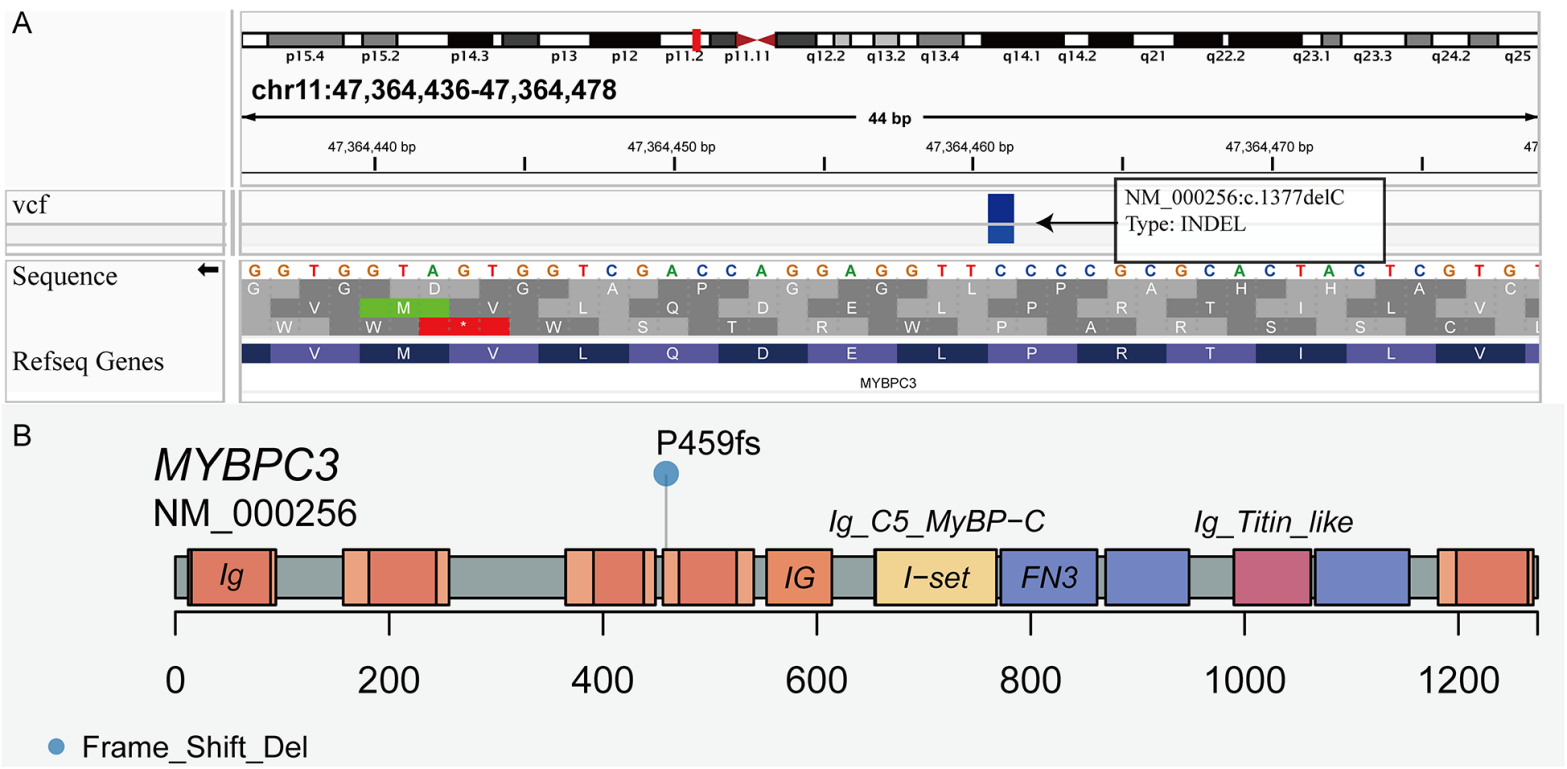
Visualization of the MyBPC3 P459fs mutation. **(A)** The frameshift mutation deleting cytosine nucleotide (c. 1377delC) was in exon 15 of *MyBPC3*. **(B)** Lollipop plot presenting the location of frameshift mutation (P459fs) in *MyBPC3*.

Original sequencing data were converted into original sequences and then stored in the FASTQ file format. We aligned the sequenced reads in FASTQ file format to hg19 (GRCh37, UCSC version) using the Burrows-Wheeler Aligner version 0.7.13 (http://bio-bwa.sourceforge.net/). Single nucleotide variants and indels were identified using Genome Analysis Toolkit Unified Genotyper in parallel with the SAMtools pipeline. Arraystar version 12 was used to annotate normal and variant alleles based on public databases (dbSNP 129, 1000 Genomes, and Human Gene Mutation Database). MyBPC3 P459fs mutation was filtered as frameshift mutation (p. P459fs), deleting cytosine nucleotide (c. 1377delC) in exon 15 of *MyBPC3* (Figure 1A, B), which was predicted as pathogenic mutation by 16 online software programs, such as SIFT, Polyphen2, LRT, MutationTaster, FATHMM, and PROVEAN, among others.

### 2.4 Echocardiography

Echocardiography was carried out using a GE Vivid 7 Ultrasound System (GE Healthcare, USA) and an EchoPAC workstation equipped with a 2–4 MHz phased-array transducer. According to the recommendations of the American Society of Echocardiography, conventional echocardiography, tissue Doppler imaging (TDI), and speckle tracking echocardiography (STE) were performed and measured.

In conventional echocardiography, LV end-diastolic dimension (LVEDD) and LV end-systolic dimension (LVESD) were measured from the parasternal long-axis view. A total of 16 segmental thicknesses (ST) were measured at end-diastole from the parasternal short-axis views. MWT was the largest ST. EF and left atrial volume (LAV) were performed using the biplane Simpson's rule in the apical four- and two-chamber views. LAV index (LAVI) was calculated with the formula (LAVI = LAV/body surface area). Peak early and late diastolic mitral inflow velocities (*E* and *A*) and *E* wave deceleration time (EDT) were measured in the apical four-chamber view using pulsed wave Doppler. Isovolumic relaxation time (IVRT) was taken as the interval time from aortic valve closure to mitral valve opening in the apical five-chamber view by continuous wave Doppler. Mitral *E*/*A* value was calculated. In tissue Doppler imaging, early diastolic velocities (*E*′) of mitral annulus were obtained from both septal and lateral sides. Mean *E*′ and *E*/*E*′ values were calculated. In speckle tracking echocardiography, longitudinal strain (LS) was assessed in the apical four-, two-, and three-chamber views in 18 segments, and circumferential strain (CS) and radial strain (RS) were assessed in parasternal short-axis at the level of the mitral valve, papillary muscle, and apical view in 18 segments. The software package automatically tracked the speckles of the LV wall and generated global and segmental LS, CS, and RS. LV twist curves were automatically built by the software. Twist degree (TD), twist rate (TR), and untwist rate (UTR) were derived and measured from twist curves by the software. TR was the positive peak in the systolic phase. UTR was the first negative peak in the early diastolic phase. Data were excluded if there were more than one segment that failed to be tracked.

### 2.5 Cell line construction

#### 2.5.1 Cell lines and cell culture

The H9C2 cell lines (ATCC, Manassas, USA) were cultured in Dulbecco's modified Eagle's medium (DMEM), supplemented with 10% fetal bovine serum (FBS), 100 U/mL penicillin and 100 *µ*g/mL streptomycin. Cells were kept in a Heracell CO_2_ incubator (Thermo Fisher Scientific, Waltham, MA, USA) in an atmosphere of 5% CO_2_ and 95% air at 37°C and passaged at a ratio of 1:3 when a confluence of 80% was reached.

#### 2.5.2 Production of *MyBPC3* and MyBPC3 P459fs recombinant lentiviral vectors

The recombinant lentiviral plasmids containing human *MyBPC3* WT and MyBPC3 P459fs mutation were constructed with the assistance of Genechem Co. (Shanghai, China). The human *MyBPC3* gene was amplified using PCR briefly. The primers were as follows: 5′-GAGGATCCCCGGGTACCGGTCGCCACCATGCCTGAGCCGGGGAAGAAG-3′ and 5′-TCCTTGTAGTCCATACCGGTCAGCTGGTCCTCCAAGGGGCGCG-3′ for *MyBPC3*, and 5′-GAGGATCCCCGGGTACCGGTCGCCACCATGCCTGAGCCGGGGAAGAAG-3′ and 5′-TCCTTGTAGTCCATACCGGTCCAGCTGGTCCTCCAAGGGCGCG-3′ for MyBPC3 P459fs. The primers were synthesized by Genechem Co. The PCR procedure was as follows: denaturing at 94℃ for 5 min; 30 cycles of denaturation at 94℃ for 30 s; annealing at 55℃ for 30 s; extension at 72℃ for 2 min; and a final extension at 72℃ for 10 min. The PCR products were verified by agarose gel electrophoresis. Then, they were purified and cloned into the GV358-eGFP vectors. Recombinant lentiviruses containing GV358-eGFP/MyBPC3 and GV358-eGFP/MyBPC3 P459fs were constructed and identified.

#### 2.5.3 Cell transfection, screening, and testing

H9C2 cells were seeded at 2 × 10^5^ cells/well in six-well plates. After the cells attached to the wall, 2.67 µL lentivirus containing GV358-eGFP/MyBPC3 with a titer of 1.5 × 10^9^ TU/mL (MOI = 20) and 3.64 µL containing GV358-eGFP/MyBPC3 P459fs with a titer of 1.1 × 10^9 ^TU/mL (MOI = 20) were added into wells separately. At 72 h, a culture medium containing 1.5 µg/mL puromycin was used to screen for ≥2 weeks to obtain stable transfected cell lines, WT cells, and P459fs cells. An empty lentiviral vector was used for negative control.

The expression of the *MyBPC3* gene was detected using real-time quantitative polymerase chain reaction (RT-qPCR). The primers of *MyBPC3* and *ACTB* genes were synthesized by GeneChem Co., and the sequences were as follows: 5′-GTCAAGTTCGACCTCAAGGTC-3′ and 5′-ACTTGGGGCACTTTCTCCCAG-3′ for *MyBPC3*, and 5′-CCCATTGAACACGGCATTG-3′ and 5′-ACGACCAGAGGCATACAGG-3′ for *ACTB*. Total RNA was extracted from WT cells and P459fs cells using Trizol reagent (Thermo Fisher Scientific, Waltham, MA, USA), and 2 µg total RNA was reverse-transcribed to cDNA using M-MLV RT kit (Promega, Madison, WI, USA.) following the manufacturer's instructions. The resulting cDNA was quantified using a SYBR Green PCR kit (Takara, Shiga, Japan). The reaction system contained 12 µL volume, including 0.6 µL cDNA template, 6 µL SYBR Premix Ex Taq, 0.3 µL primers, and 5.1 µL ddH_2_O. The thermocycling conditions were as follows: 5 s at 95°C, followed by 50 cycles of 95°C for 5 s, 60°C for 50 s. The mRNA expression level of *ACTB* was used for normalization. The threshold cycle (Ct) value was recorded, and the data were analyzed using the comparative 2-ΔΔCt method.

### 2.6 Cell imaging using super-resolution imaging

Cell imaging was performed using a GI-SIM system that was equipped with an invented fluorescence microscope (IX83, Olympus), a laser combiner of two laser beans (488 nm laser [500 mW], Coherent Genesis Max and 560 nm laser [500 mW], MPB Communications), an acousto-optic tunable filter (AOTF, AA Quanta Tech), a phase modulator consisting of a polarization beam splitter, a ferroelectric spatial light modulator (SLM; Forth Dimension Displays, SXGA-3DM), an achromatic half-wave plate (HWP; Bolder Vision Optik), and a polarization rotator (Meadowlark). The beams passed through the AOTF, expanded to 15 mm, and were sent to the phase modulator. The beams were diffracted by the SLM, polarized by the polarization rotator, and then relayed into the back pupil of the objective (UPLSAPO100XS, Olympus). Thus, the 0 and ±1 diffraction beams intersected at the interface between the coverslip and the sample at an angle exceeding the critical angle for total internal reflection. Meanwhile, the fluorescence was collected with the same objective and relayed onto an sCMOS camera (Hamamatsu, Orca Flash 4.0 v3) to acquire the raw images. The exposure time for each raw image was 10 ms at 20 W/cm^2^ light intensity.

### 2.7 Metabolomic profiles

P459fs cells and WT cells were used in metabolomic and proteomic profiles. Cells were mixed and suspended with 300 μL methanol. The mixtures were subjected to three freeze–thaw cycles (liquid nitrogen for 5 min, −0℃ ice for 5 min, −37℃ water for 5 min). Then the supernatants were collected after centrifuging (13,000 rpm, 4℃, 15 min), which were used for liquid chromatography–mass spectrometry (LC/MS) analysis. The analytical system was an LC–ESI–MS/MS system (HPLC, Shim-pack UFLC SHIMADZU CBM30A system; MS, Applied Biosystems 6,500 Q TRAP). The analytical conditions were as follows: column, Waters ACQUITY UPLC HSS T3 C18 (1.8 µm, 2.1 mm × 100 mm); solvent system, 0.04% acetic acid; gradient program, 95:5 V/V at 0 min, 5:95 V/V at 11.0 min, 5:95 V/V at 12.0 min, 95:5 V/V at 12.1 min, 95:5 V/V at 15.0 min; flow rate, 0.40 mL/min; temperature, 40°C; injection volume, 2 μL. The effluent was alternatively connected to an ESI-triple quadrupole-linear ion trap (Q TRAP)-MS. The following scans by ESI-Q TRAP-MS/MS were operated in a positive ion mode and controlled by using Analyst 1.6 software (AB Sciex). The parameters were as follows: ion source, turbo spray; source temperature, 500°C; ion spray voltage (IS), 5,500 V; ion source gas I (GSI), gas II (GSII), and curtain gas (CUR) were set at 55, 60, and 25 psi, respectively; the collision gas was high. Instrument tuning and mass calibration were performed with 10 and 100 μmol/L polypropylene glycol solutions in QQQ and LIT modes, respectively.

Filtering and aligning of the raw data were performed with Mass Profiler Professional software version 13.0 (Agilent, USA). Background noise and unrelated ions were removed using the Molecular Feature Extraction (MFE) tool in the Mass Hunter Qualitative Analysis software B.06.00 (Agilent, USA). The multivariate analyses were performed using SIMCA-PC software 12.0.1.0 (Umetrics, Sweden) to generate a partial least squares discriminant analysis (PLS-DA) model with all the variables; quality controls (QCs) were predicted into this model. These data were then represented in a hierarchical condition tree. Their identities were confirmed by comparing the fragments that were obtained with the structure of the proposed compound in the MS/MS spectra in a public database (METLIN: https://metlin.scripps.edu/metabolites_list.php) or against commercially available standards. The Wilcoxon rank-sum test was used to assess the metabolites with significant differences in abundance between P459fs cells and WT cells.

### 2.8 Proteomic profiles

Cells were added and suspended with a proteinase comparable buffer. Cells were lysed using a triple repeat of the freeze–thaw method described above. Then, the peptides were labeled with 6-plex TMTs kits (Thermo Fisher, USA) according to the manufacturer's protocol. For labeling, each TMT reagent (defined as the amount of reagent required to label 100μg of peptides) was thawed and reconstituted in acetonitrile. The peptides were incubated for 2 h at room temperature and pooled for LC-MS/MS analysis. The analytical system was an EASY-nLC 1,200 coupled with Q Exactive HF. The analytical conditions were as follows: column, reverse column (3 µm, 75 µm × 20 cm); solvent system, buffer A (0.1% formic acid/100% H_2_O) and buffer B (0.1% formic acid/100% acetonitrile); gradient program, 96% buffer A and 4% buffer B at 0 min, 92% buffer A and 8% buffer B at 8 min, 78% buffer A and 22% buffer B at 58 min, 68% buffer A and 32% buffer B at 70 min, and hold 10% buffer A and 90% buffer B for 5 min; flow rate, 0.30 mL/min; spray voltage, 2.0 kV; temperature, 320°C; survey scan, 300–1,600 m/z; dynamic exclusion duration, 40 s.

The MS data were analyzed using Thermo Proteome Discoverer (2.2.0.388) and The Uniprot_protein_human or Rat database (update-20171001). The MS analysis was operated in a positive ion mode. The search parameters were as follows: enzyme, trypsin; maximum missed cleavages, two; MS/MS tolerance, 10 ppm; MS/MS peaks, 20 mDa, fixed modifications, carbamidomethyl (cysteine), and TMT (protein N-term); variable modifications, TMT (lysine), biotin (lysine), and oxidation (methionine). The false discovery rate (FDR) was set as 1%, and at least one peptide was required for identification.

### 2.9 Statistical analysis

For human participants, independent two-sample *t*-tests or chi-square tests were performed to assess the inter-group differences in demographic data, clinical characteristics, and echocardiographic data using the SPSS 16.0 statistical software package (SPSS, Chicago, IL). Statistical significance was set at *p* < 0.05.

For rat cell lines, the differences in myocardial fiber length and positive myocardial fibers between P459fs cells and WT cells were evaluated with independent two-sample *t*-tests and chi-square tests. Differentially expressed metabolites were defined as metabolites with FDR < 0.05 and variable importance in the projection (VIP) value > 1. Differentially expressed proteins were defined as proteins with FDR <0.05 and ≥1.5-fold changes. An adjusted *p*-value < 0.05 was considered indicative of significantly enriched Kyoto Encyclopedia of Genes and Genomes (KEGG) pathways.

## 3 Results

### 3.1 HCM patients carrying MyBPC3 P459fs mutation exhibit LV diastolic and systolic dysfunction

**Figure 2:**
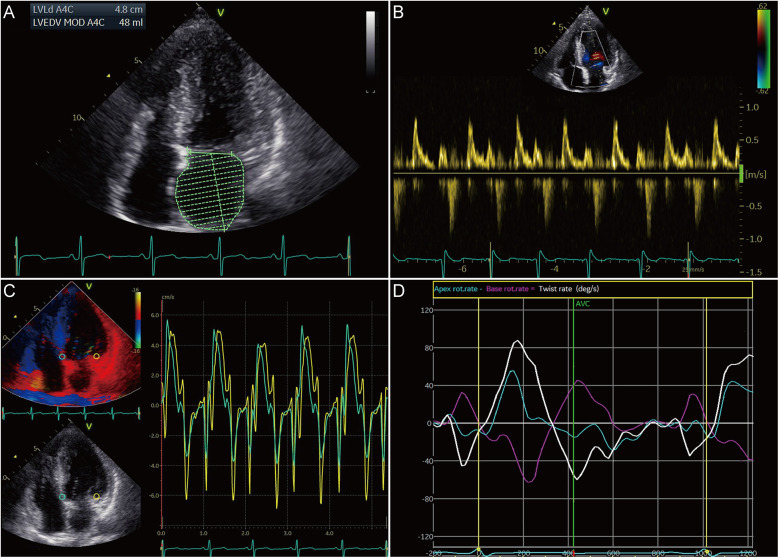
Diastolic dysfunction in HCM patients carrying the MyBPC3 P459fs mutation. **(A)** MyBPC3-P459fs HCMs expressed increased LAVI, which was performed by the biplane Simpson's rule in the apical four- and two-chamber views. **(B,C)** There were increased *E*/*E*′ ratio, decreased mean *E*′ value, and decreased *A*′ value in MyBPC3-P459fs HCMs. **(D)** Decreased untwisting and twisting rates were detected in MyBPC3-P459fs HCMs. MyBPC3-P459fs HCMs, hypertrophic cardiomyopathic patients carrying MyBPC3 P459fs mutation; LAVI, left atrial volume index; *E*′ and *A*′, velocity of the mitral annulus in the early and late diastolic phase.

**Figure 3:**
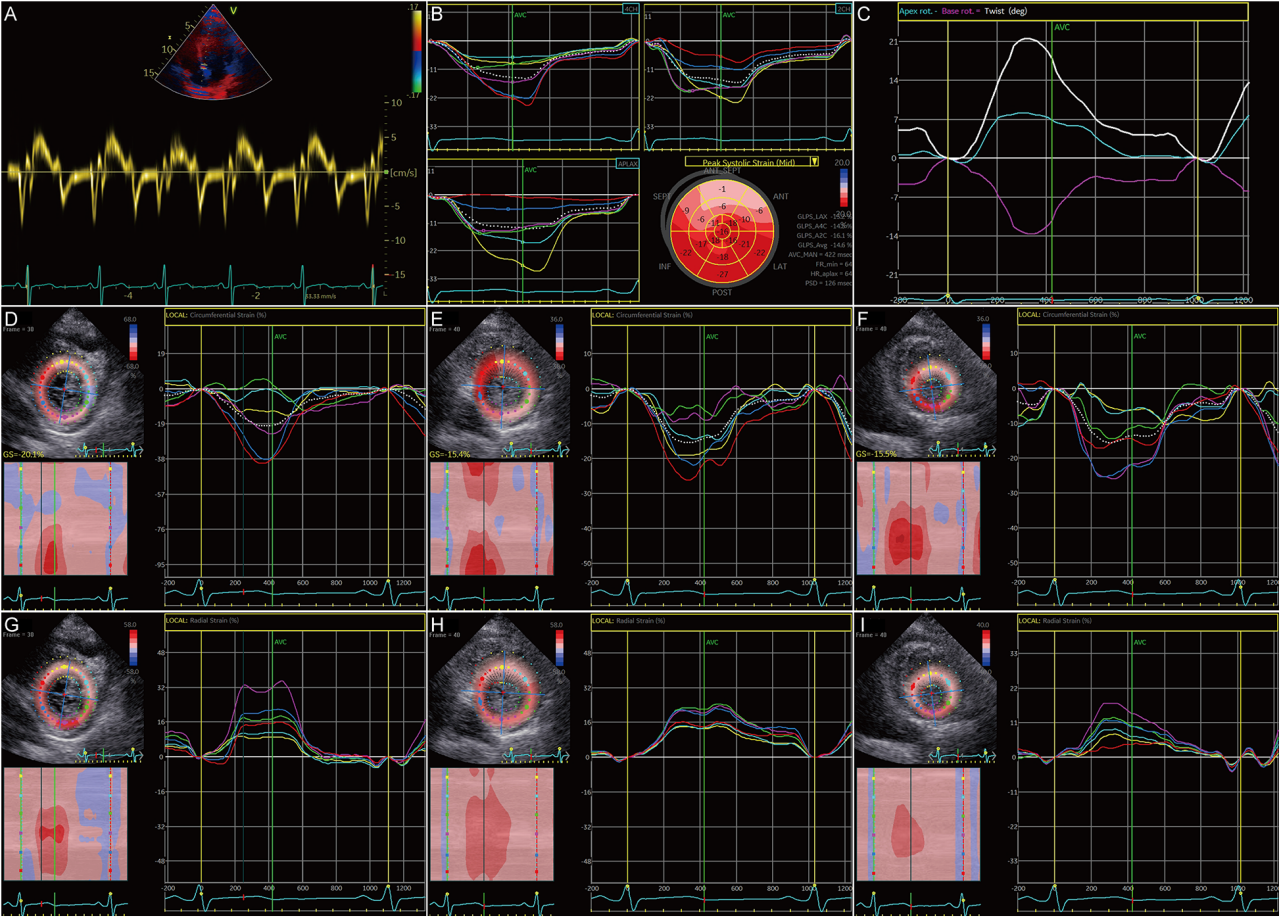
Systolic dysfunction in HCM patients carrying the MyBPC3 P459fs mutation. **(A)** MyBPC3-P459fs HCMs showed decreased S’. **(B)** GLS was decreased in MyBPC3-P459fs HCMs. **(C)** The twist degree was decreased in MyBPC3-P459fs HCMs. **(D–F)** MyBPC3-P459fs HCMs showed decreased CS at the level of the mitral valve, papillary muscle, and apical view. (**G–I**) MyBPC3-P459fs HCMs showed decreased RS at the level of the mitral valve, papillary muscle, and apical view. MyBPC3-P459fs HCMs, hypertrophic cardiomyopathy patients carrying MyBPC3 P459fs mutation; *S*′, velocity of the mitral annulus in systolic phase; GLS, global longitudinal strain; CS, circumferential strain; RS, radial strain.

To ascertain the effects of MyBPC3 P459fs mutation on LV diastolic and systolic function of HCM patients, five MyBPC3-P459fs HCMs and 10 HCs were recruited, who were identified either by WES or panel sequencing. MyBPC3-P459fs HCMs exhibited significant LV diastolic dysfunction, characterized as higher LAVI value (38.92 ± 14.46 vs. 23.98 ± 7.33, *p* = 0.018), higher *E*/*E*′ ratio (14.99 ± 2.51 vs. 7.83 ± 1.17, *p* < 0.001), lower mean *E*′ (6.00 ± 0.25 cm/s vs. 10.30 ± 2.12 cm/s, *p* = 0.001), lower mean *A*′ value (6.70 ± 0.91 cm/s vs. 9.18 ± 1.23 cm/s, *p* = 0.002), and lower UTR (−61.9 ± 27.02°/s vs. −108.93 ± 40.32°/s, *p* = 0.036) compared to HCs (Figure 2A–D) (Supplementary Table S1). MyBPC3-P459fs HCMs also showed LV systolic dysfunction, which was manifested as lower S’ value (6.10 ± 0.74 cm/s vs. 9.90 ± 1.94 cm/s, *p* = 0.001), lower global and segmental LS (−12.96 ± 1.37% vs. −23.20 ± 1.26%, *p* < 0.001), CS (−19.94 ± 2.76% vs. −25.43 ± 1.88%, *p* = 0.001) and RS (25.70 ± 4.45% vs. 38.53 ± 8.86%, *p* = 0.010), and lower TD (10.89 ± 7.34° vs. 24.98 ± 8.72°, *p* = 0.009) compared to HCs (Figures 3A–I, 2D) (Supplementary Tables S1, S2). There were no significant differences between MyBPC3-P459fs HCMs and HCs in demographic and clinical data, except for NT-proBNP (755.40 ± 578.01 pg/mL vs. 52.40 ± 19.83 pg/mL, *p* < 0.001) (Supplementary Table S3).

### 3.2 Myocardial cells with MyBPC3 P459fs mutation showed myocardial fibers disarray

**Figure 4:**
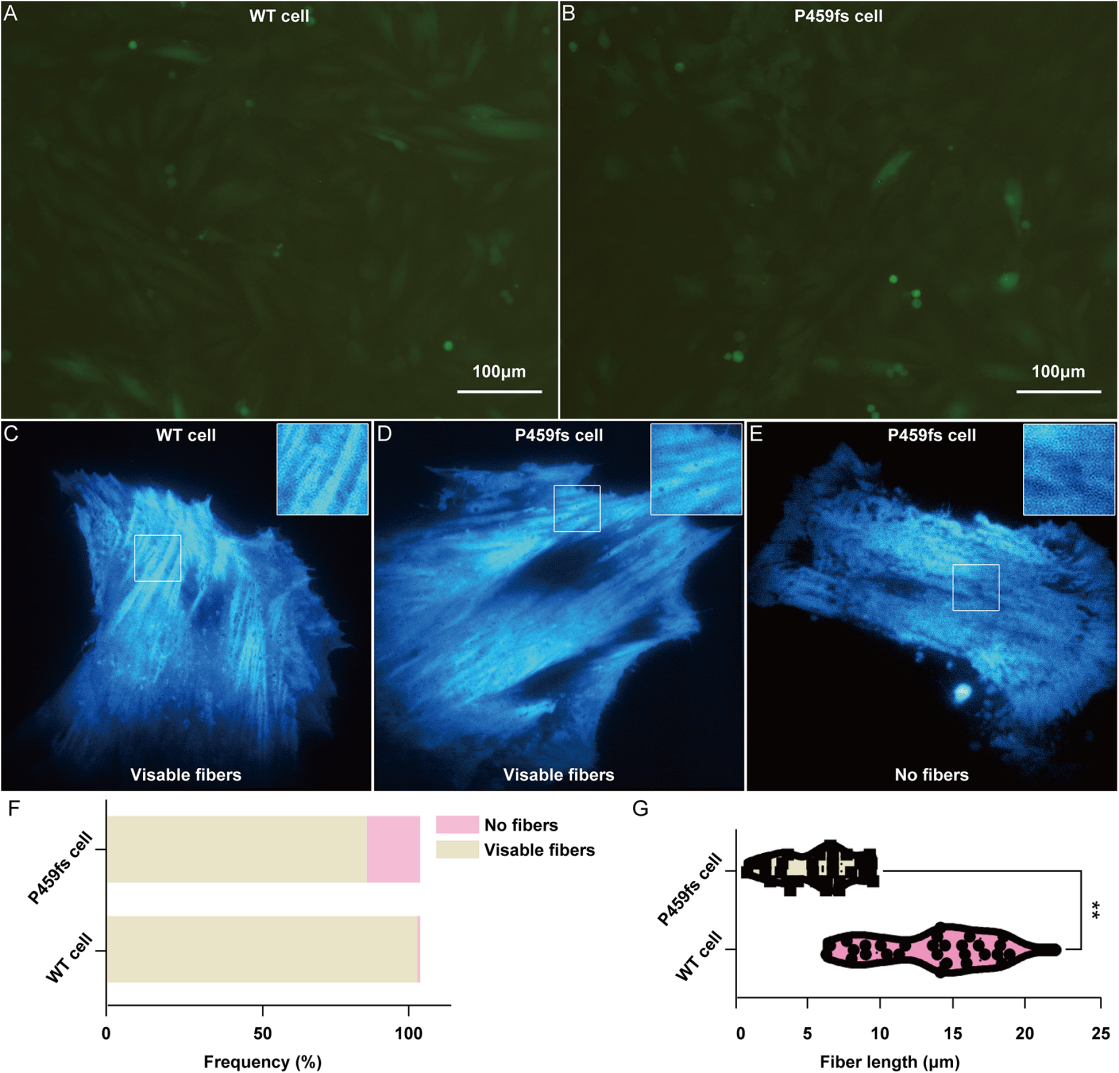
Myocardial fibers disarray in P459fs cells. **(A,B)** The stable cell lines that expressed MyBPC3 P459fs mutation and *MyBPC3* wild type (×100). **(C–F)** P459fs cells and WT cells formed myocardial fibers in the cytoplasm. P459fs cells formed fewer myocardial fibers or no myocardial fibers. **(G)** Compared to WT cells, P459fs cells had shorter myocardial fibers. P459fs cells, H9C2 cells with MyBPC3 P459fs mutation; WT cells, H9C2 cells with *MyBPC3* wild type.

To further identify the role of MyBPC3 P459fs mutation in myocardial cells, we constructed recombinant lentiviral vectors expressing MyBPC3 P459fs mutation and its WT counterpart, packaged virus in 293T cells, and infected H9C2 cells with the above lentivirus. We successfully obtained the stable cell lines that expressed MyBPC3 P459fs mutation (P459fs cells), *MyBPC3* WT cells, and empty vector (EV cells) (Figure 4A, B). Relative to EV cells, the abundance of MyBPC3 mRNA was determined in both WT and P459fs cells, indicating the success of stable infection.

We next sought to understand the effects of MyBPC3 P459fs mutation on myocardial fiber arrangements in P459fs cells and WT cells. First, there were three independent biological replicates, with 100 P459fs cells and 100 WT cells for each biological replicate. We found that the myocardial fibers of P459fs cells and WT cells formed in the cytoplasm, with their respective mutant and WT counterparts (Figure 4C–E), whereas approximately 15% of P459fs cells could not form myocardial fibers (Figure 4F). In addition, we measured the mean length of myocardial fibers in each cell, with 20 P459fs cells and WT cells, respectively. The distributions of mean length in these cells are illustrated in Figure 4G. It showed that the length of myocardial fibers in P459fs cells was significantly shorter than that of WT cells. Taken together, possibly for the first time, the super-resolution imaging results suggested that MyBPC3 P459fs mutation might weaken the longitudinal contraction of myocardial fibers, which had a high clinical relevance.

### 3.3 Metabolomic files of MyBPC3 P459fs mutation revealed metabolic disturbance

An energetic imbalance was observed in the HCM mutant patients, which contributed to the cardiac dysfunction (17). A deeper understanding of the metabolic and energetic alterations underlying cardiac dysfunction is required. Metabolomic profiles were analyzed to reveal the potential pathways of MyBPC3 P459fs mutation in myocardial dysfunction.

**Figure 5:**
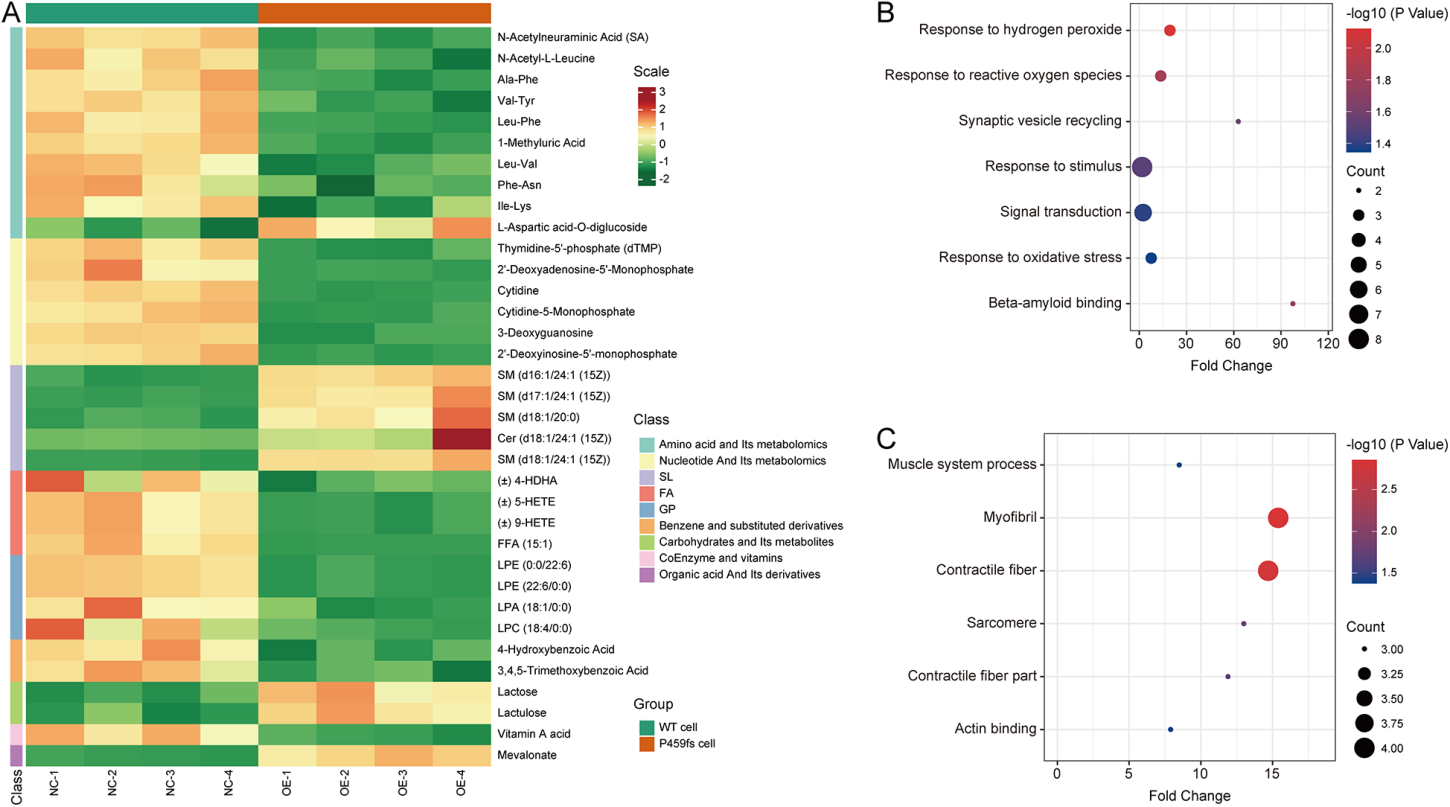
Metabolomic and proteomic characteristics of P459fs cells. **(A)** Cluster analysis of metabolomic profiles between P459fs cells and WT cells. Compared to WT cells, the metabolites in P459fs cells were significantly increased in sphingomyelin, lactose, lactulose, and mevalonate, while significantly decreased in amino acids and their derivatives, nucleotides and their derivatives, fatty acids, phospholipid, and vitamin A. **(B)** Pathways of proteomic profiles of the upregulated proteins between P459fs cells and WT cells. The proteins enriched in response to oxidative stress and amyloid binding. **(C)** Pathways of proteomic profiles of the downregulated proteins between P459fs cells and WT cells. The significant network was actin binding capacity. P459fs cells, H9C2 cells with MyBPC3 P459fs mutation; WT cells, H9C2 cells with *MyBPC3* wild type.

A total of 727 metabolites were identified. On this basis, there were nine significantly upregulated metabolites and 26 significantly downregulated metabolites. Compared to WT cells, the upregulated metabolites in P459fs cells were enriched in sphingomyelin, lactose, lactulose, and mevalonate, while the downregulated metabolites were enriched in amino acids and their derivatives, nucleotides and their derivatives, fatty acids, phospholipid, and vitamin A (Figure 5A) (Supplementary Table S4). These findings suggested that there were structural and functional disturbances in P459fs cells, e.g., sphingomyelin, phospholipid, and nucleotides in mitochondrial membrane integrity (18–20), mevalonate and vitamin A in myocardial remodeling (21, 22), as well as lactose, lactulose, fatty acids, and amino acids and their derivatives in myocardial energy metabolism (23).

### 3.4 Quantitative proteomics revealed key differences by MyBPC3 P459fs mutation

In addition, we utilized TMTs labeled proteomic profiles to identify the proteins altered by MyBPC3 P459fs mutation. In the proteomic analysis, a total of 4,059 proteins were identified. A 1.3-fold change was set as the cutoff threshold. We observed significant differences in 12 upregulated proteins (Grn, Vps29, Itm2b, RGD1310127, Rab43, Nol3, Olr1121, Hba1, Dap1, C2cd2, Pld2, and uncharacterized protein) and 13 downregulated proteins (Myh3, Kdelc2, Myl4, Skiv2l, Cavin4, Synpo2l, Srpx, Babam1, Ptgr2, Trip10, Ppox, Ranbp6, and uncharacterized protein) when comparing P459fs cells to WT cells (Supplementary Table S5). GO analysis revealed that the upregulated proteins were enriched in response to oxidative stress and amyloid binding (Figure 5B). Notably, the actin binding capacity was impaired by the MyBPC3 P459fs mutation (Figure 5C). These findings suggested that several pathogenic pathways were involved in MyBPC3-P459fs mutation, including oxidative stress (24), inflammation related to amyloid (25), and actin binding capacity (26).

## 4 Discussion

Although *MyBPC3* mutations were the most common genetic cause of familial HCM, the links and pathways from the inciting mutation to cardiac dysfunction had been elusive. We previously reported cytosine nucleotide deletion in exon 15 of *MyBPC3* that could cause a pathogenic MyBPC3 P459fs mutation (27). Here, we took advantage of super-resolution imaging and multi-omics data to elucidate how MyBPC3 P459fs mutation led to cardiac dysfunction and myocardial fiber disarray. Importantly, we found that MyBPC3 P459fs mutation was associated with mitochondrial membrane integrity, myocardial remodeling, and myocardial energy metabolism in metabolomic profiles, and oxidative stress, inflammation, and actin binding capacity in proteomic profiles.

In this study, MyBPC3-P459fs HCMs exhibited LV diastolic and systolic dysfunction, suggesting that myocardial deformation was impaired in MyBPC3-P459fs HCMs. The myocardial fibers formed two helices of opposing direction that were electrically and mechanically interconnected, resulting in myocardial deformation becoming an interplay of all fibers throughout four major components—longitudinal, circumferential, radial, and shear (twist)—which allowed to transform a fiber shortening of only 15% into a 60% change in LV volume (28, 29). Any pathology of myocardial fibers could influence all four strain components and thus led to LV diastolic and systolic dysfunction. Suay-Corredera et al. pointed out that frameshift mutation in the *MyBPC3* gene could result in truncated polypeptides, which were more prone to lead to cMyBP-C haploinsufficiency in HCM (7). Protein haploinsufficiency could result in converging misregulation of contraction and relaxation of myocardial fibers. Toepfer et al. found that frameshift mutation in the *MyBPC3* gene and RNAi silencing of the *MyBPC3* gene could cause comparable abnormalities in myocardial contraction and relaxation (8). The evidence above supported our findings of LV diastolic and systolic dysfunction in MyBPC3-P459fs HCMs.

Our study revealed that, for the first time in live P459fs cells, myocardial fibers were disarrayed, manifesting as fewer and shorter myocardial fibers in the cytoplasm. There was evidence that myocardial fiber disarray was involved in the whole pathological course of HCM. Adverse clinical outcomes of HCM, such as cardiac arrhythmia, heart failure, and sudden cardiac death, were thought to correlate to myocardial fiber disarray (30). *MyBPC3* frameshift mutation showed alterations in RNA splicing or protein stability, both of which could lead to cMyBP-C haploinsufficiency (31). Our result suggested that MyBPC3 P459fs mutation and *MyBPC3* knockout could cause comparable abnormalities in myocardial fibers. Jiang et al. showed that mice with *MyBPC3* knockout had increased numbers of fragile mononuclear myocardial cells, which could promote myocardial cell death and fibrosis (32). Garcia-Canadilla et al. found that mice with *MyBPC3* knockout showed fewer myocardial fibers, which were more widely arranged in the anterior, septal, lateral, and inferior walls (30). The abnormality in myocardial fibers in *MyBPC3* frameshift mutation or *MyBPC3* knockout could cause dysfunction in myocardial contraction and relaxation. Taylor et al. demonstrated that phospho-mimetic *MyBPC3* mutation could result in global myofiber disarray, such as reduced length, increased width, and loss of helicity, which were associated with reduced contraction and altered torsional mechanics (33). Ma et al. showed that *MyBPC3* knockout exhibited calcium transient abnormalities, impaired force-development kinetics, and deficient contraction (34). Our result showed that MyBPC3 P459fs mutation could result in fewer and shorter myocardial fibers. These findings, together with our results, confirmed that *MyBPC3* frameshift mutation, e.g., MyBPC3 P459fs mutation, could cause myocardial fiber disarray and finally result in impaired myocardial contraction and relaxation.

Metabolomic profiles revealed that there was dysregulation of sphingomyelin, phospholipid, and nucleotides in P459fs cells, representing decreased mitochondrial membrane integrity. Myocardial cells with hypertrophy and fibrosis were derived from loss of mitochondrial membrane integrity and were accompanied by an increase in membrane permeability and myocardial cell apoptosis (35, 36). Meanwhile, Ranjbarvaziri et al. found that myocardial samples from HCM patients showed decreased phospholipids, increased sphingolipids, and decreased nucleotides (37). Our findings also showed that mevalonate and vitamin A were disturbed in P459fs cells, which were involved in myocardial remodeling. Mevalonate affected various metabolism pathways, such as protein synthesis and degradation, intracellular signaling, cell growth, and differentiation or death (38). Chen et al. found that mevalonate served an important role in ventricular remodeling, especially in pressure overload-induced cardiac hypertrophy and associated diastolic dysfunction (39). Evidence had emerged that vitamin A was deeply involved in regulating cardiac regeneration, cardiac function, and pathological hypertrophy (40). Azevedo et al. revealed that vitamin A-deficient rats showed reduced systolic function (41). In our study, myocardial energy metabolism appeared altered in lactose, lactulose, fatty acids, and amino acids and their derivatives in P459fs cells. Van der Velden et al. demonstrated a metabolic shift from fatty acid oxidation to glucose oxidation in HCM patients (42). Jansen et al. found that in HCM patients with severe phenotypes, several pathways were dysregulated, including lysine, histidine, lysine, acylcarnitine, purine, and protein hydrolysis (43). The above findings were consistent with our results that a series of metabolic disturbances ultimately led to structural and functional disorders in HCM.

In our study, proteomic analysis showed that the expression levels of multiple proteins changed in P459fs cells, which were involved in oxidative stress, inflammation related to amyloid, and actin binding capacity. Cohn et al. identified p53 activation, oxidative stress, and cytotoxicity in MyBPC3-R502W+/– cardiomyocytes (24). Wijnker et al. demonstrated that *MyBPC3* mutations could affect oxidative stress and were associated with diastolic dysfunction, fibrosis, cardiac hypertrophy, and arrhythmias, which are all hallmarks of HCM (44). Moreover, consistent evidence had been gathered through other studies showing that inflammation related to amyloid was involved in the pathological process of HCM. Fang et al. found that serum amyloid was significantly increased and was associated with diastolic dysfunction in HCM patients (45). Jang et al. showed that amyloid had adverse effects on cell viability and mitochondrial function in myocardial cells, which deteriorated diastolic dysfunction and increased left ventricular wall thickness (46). In P459fs cells, we found that downregulated proteins were enriched in actin binding capacity, which was involved in the maintenance of the structure and function in actin filaments. Da'as et al. found that there was a significant downregulation of the actin-filament-based process and an impaired actin cytoskeleton organization in the zebrafish MyBPC3 HCM model (47). Hassoun et al. demonstrated that *MyBPC3* mutation could cause perturbed myosin dynamics and disrupted actin inhibitory regulation, as well as leading to increased Ca^2+^ sensitivity in HCM (48). These findings and our results confirmed that *MyBPC3* mutations, such as MyBPC3 P459fs mutation, could provide a greater understanding of the structural and functional disorders in HCM.

Several limitations should be noted. First, due to the rarity of the MyBPC3 P459fs mutation, our study included only five HCM patients with this mutation, all of whom were of Chinese descent. In addition, further studies with larger sample sizes and diverse populations were still needed to validate our findings. Second, although H9C2 cells were recognized as the most popular cell lines in myocardial research, induced pluripotent stem cell-derived cardiomyocytes should be utilized to gain more information on HCM patients with MyBPC3 P459fs mutation and explore the deeper mechanisms of myocardial dysfunction on HCM with MyBPC3 P459fs mutation in the future.

## 5 Conclusions

In summary, there were significant disorders in LV diastolic and systolic function and myocardial fibers disarray in clinical HCM patients with P459fs mutation. Further, metabolomic and proteomic changes in P459fs cells suggested mitochondrial membrane integrity, myocardial remodeling, myocardial energy metabolism, oxidative stress, inflammation, and actin binding capacity as potential pathways of structural and functional disorders in HCM with P459fs mutation. These findings bridge the gap between human data and cell models in HCM with the P459fs mutation, potentially contributing to more effective individualized treatment. As the first study of its kind, further research and replication are needed to validate these results.

## Data Availability

The datasets presented in this study can be found in online repositories. The names of the repository/repositories and accession number(s) can be found in the article/Supplementary Material.
