# Supplementary material for: Myocardial dysfunction caused by MyBPC3 P459fs mutation in hypertrophic cardiomyopathy: evidence from multi-omics approaches and super-resolution imaging
Source: Front Cardiovasc Med. 2025 Feb 27;12:1529921. doi: 10.3389/fcvm.2025.1529921 (PMC11903464; doi:10.3389/fcvm.2025.1529921)
Supplement: Supplementary file 1 [file Datasheet1.pdf]

Supplementary Materials

1 Supplementary Tables

Table S1 Echocardiographic differences between MyBPC3-P459fs HCMs and HCs.

Variables	MyBPC3-P459fs HCMs (n = 5)	HCs (n = 10)	T	P value
LVEDD (mm)	41.38±6.17	46.26±3.47	-1.989	0.068
LVESD (mm)	27.64±5.89	29.14±3.62	-0.616	0.548
EF (%)	67.00±4.47	64.60±3.24	1.196	0.253
LAVI	38.92±14.46	23.98±7.33	2.706	0.018
E (m/s)	0.90±0.13	0.79±0.14	1.372	0.193
A (m/s)	0.70±0.10	0.81±0.12	-1.603	0.133
E/A	1.30±0.27	1.01±0.26	1.971	0.070
mean E' (cm/s)	6.00±0.25	10.30±2.12	-4.429	0.001
mean S' (cm/s)	6.10±0.74	9.90±1.94	-4.163	0.001
mean A' (cm/s)	6.70±0.91	9.18±1.23	-3.972	0.002
E/E'	14.99±2.51	7.83±1.17	7.690	<0.001
GLS (%)	-12.96±1.37	-23.20±1.26	14.438	<0.001
GCS (%)	-19.94±2.76	-25.43±1.88	4.578	0.001
GRS (%)	25.70±4.45	38.53±8.86	-3.013	0.010
Twist degree (º)	10.89±7.34	24.98±8.72	-3.094	0.009
Twisting rate (º/s)	66.72±29.71	101.42±43.62	-1.589	0.136
Untwist rate (º/s)	-61.9±27.02	-108.93±40.32	2.337	0.036

MyBPC3-P459fs HCMs, hypertrophic cardiomyopathic patients carrying MyBPC3 P459fs mutation; HCs, healthy controls; LVEDD, left ventricular end-diastolic dimension; LVESD, left ventricular end-systolic dimension; EF, ejection fraction; LAVI, left atrial volume index; E and A, velocity of mitral valve in early and late diastolic phase; E’, A’ and S’, velocity of mitral annulus in early and late diastolic phase and systolic phase; GLS, global longitudinal strain; GCS, global circumferential strain; GRS, global radial strain.

Table S2 The difference of segmental strains between MyBPC3-P459fs HCMs and HCs.

Variables	MyBPC3-P459fs HCMs<br>n = 5	HCs<br>n = 10	T	P value
LS (%)				
4Ch-LS	-12.42±3.12	-22.18±2.27	6.966	<0.001
4Ch-BasSeptLS	-11.20±3.74	-18.46±3.32	3.836	0.002
4Ch-MidSeptLS	-10.01±5.81	-18.77±2.93	3.958	0.002
4Ch-ApiSepLS	-11.93±8.34	-26.87±4.06	4.760	<0.001
4Ch-ApiLatLS	-7.99±4.78	-27.47±5.47	6.755	<0.001
4Ch-MidLatLS	-13.05±3.51	-22.04±3.86	4.367	0.001
4Ch-BasLatLS	-20.32±5.33	-19.46±3.72	-0.369	0.718
2Ch-LS	-13.90±1.65	-22.82±4.27	4.442	0.001
2Ch-BasSeptLS	-22.93±2.87	-19.38±2.67	-2.375	0.034
2Ch-MidSeptLS	-18.28±2.03	-20.47±3.20	1.386	0.189
2Ch-ApiSepLS	-12.62±1.23	-27.14±5.98	5.278	<0.001
2Ch-ApiLatLS	-9.26±2.92	-28.01±8.19	4.886	<0.001
2Ch-MidLatLS	-9.09±2.80	-21.37±7.46	3.500	0.004
2Ch-BasLatLS	-11.23±3.74	-20.56±4.37	4.066	0.001
3Ch-LS	-12.57±2.43	-24.59±3.15	7.453	<0.001
3Ch-BasPostLS	-24.34±2.27	-22.55±4.25	-0.872	0.399
3Ch-MidPostLS	-18.89±2.70	-21.78±3.81	1.505	0.156
3Ch-ApiPostLS	-11.80±5.30	-26.93±6.27	4.615	<0.001
3Ch-ApIAntSeptLS	-4.83±3.85	-29.86±8.76	6.017	<0.001
3Ch-MidAntSeptLS	-6.55±1.92	-23.92±5.11	7.244	<0.001
3Ch-BasAntSeptLS	-9.03±4.59	-22.51±5.99	4.400	0.001
CS (%)				
Bas-CS	-20.14±2.42	-25.72±3.49	3.187	0.007
Bas-AntSeptCS	-22.34±5.49	-31.04±4.73	3.192	0.007
Bas-AntCS	-13.89±5.86	-23.18±7.88	2.318	0.037
Bas-LatCS	-15.45±1.74	-19.91±5.36	1.786	0.097
Bas-PostCS	-19.15±7.75	-21.15±4.90	0.616	0.549
Bas-InfCS	-22.01±10.28	-24.82±8.09	0.582	0.570
Bas-SeptCS	-27.97±7.89	-34.22±9.20	1.294	0.218
Mid-CS	-20.32±3.75	-24.48±3.60	2.084	0.057
Mid-AntSeptCS	-22.70±1.41	-31.34±8.01	2.352	0.035
Mid-AntCS	-18.98±1.69	-23.55±6.54	1.509	0.155
Mid-LatCS	-17.53±7.23	-18.34±4.82	0.261	0.798
Mid-PostCS	-19.19±10.99	-17.38±5.84	-0.422	0.680
Mid-InfCS	-21.17±8.09	-24.70±8.73	0.755	0.464
Mid-SeptCS	-22.35±3.82	-31.58±7.34	2.606	0.022
Api-CS	-19.36±3.92	-26.07±4.18	2.987	0.010
Api-AntSeptCS	-16.10±7.96	-23.88±5.02	2.336	0.036
Api-AntCS	-17.3±12.82	-24.21±10.04	1.151	0.271
Api-LatCS	-20.49±10.54	-24.42±8.60	0.776	0.452
Api-PostCS	-23.18±6.86	-27.87±6.42	1.306	0.214
Api-InfCS	-21.77±4.28	-29.09±4.85	2.853	0.014
Api-SeptCS	-17.31±8.48	-26.97±5.13	2.778	0.016
RS (%)				
Bas-RS	35.03±5.78	39.57±25.08	-0.393	0.701
Bas-AntSeptRS	17.92±5.35	36.49±19.27	-2.079	0.058
Bas-Ant-RS	23.22±4.12	39.56±25.18	-1.415	0.181
Bas-LatRS	38.42±8.16	39.37±26.66	-0.077	0.940
Bas-PostRS	52.9±11.32	41.83±27.50	0.852	0.409

Bas-InfRS	46.83±7.82	42.12±31.70	0.322	0.753
Bas-SeptRS	30.87±6.99	38.04±23.65	-0.653	0.525
Mid-RS	27.35±4.19	39.65±16.39	-1.623	0.129
Mid-AntSeptRS	17.52±3.00	38.09±15.37	-2.912	0.012
Mid-AntRS	19.29±3.58	38.38±15.62	-2.651	0.020
Mid-LatRS	30.59±7.34	39.99±19.21	-1.040	0.317
Mid-PostRS	37.96±6.34	39.61±16.95	-0.206	0.840
Mid-InfRS	33.77±7.43	40.57±17.53	-0.818	0.428
Mid-SeptRS	24.94±4.53	41.27±19.09	-1.853	0.087
Api-RS	14.73±9.44	36.38±14.21	-3.057	0.009
Api-AntseptRS	12.91±9.40	34.88±11.24	-3.748	0.002
Api-AntRS	12.01±11.02	32.26±10.80	-3.400	0.005
Api-LatRS	13.83±10.47	32.50±12.74	-2.820	0.014
Api-PostRS	16.38±9.58	34.85±14.75	-2.521	0.026
Api-InfRS	17.56±8.82	39.54±17.90	-2.559	0.024
Api-SeptRS	15.66±8.87	44.24±24.69	-2.470	0.028

MyBPC3-P459fs HCMs, hypertrophic cardiomyopathic patients carrying MyBPC3 P459fs mutation; HCs, healthy controls; LS, longitudinal strain; CS, circumferential strain; RS, radial strain; 4Ch, 2Ch and 3Ch, apical four-, two-, and three-chamber view; Bas, basal; Mid, middle; Api, apical; Ant, anterior; Sept, septal; Inf, inferior; Post, posterior; Lat, lateral.

Table S3 The difference of clinical data between MyBPC3-P459fs HCMs and HCs.

Variables	MyBPC3-P459fs HCMs (n = 5)	HCs (n = 10)	T/χ2	P value
Age (years)	50.60±6.19	52.00±7.36	-0.364	0.722
Male (n, %)	2 (40.0)	4 (40.0)	0.000	0.713
Heigh (m)	1.62±0.09	1.64±0.08	-0.386	0.706
Weight (kg)	62.40±13.96	63.25±11.82	-0.124	0.903
BMI	23.58±4.04	23.32±2.41	0.159	0.876
BSA (m2)	1.64±0.22	1.66±0.20	-0.194	0.849
Smoking (n, %)	2 (40.0)	3 (30.0)	0.150	0.566
Drinking (n, %)	0 (0.0)	1 (10.0)	0.536	0.667
SBP (mmHg)	122.40±10.43	123.40±11.71	-0.176	0.863
DBP (mmHg)	78.20±6.57	80.07±7.43	-0.475	0.643
HR (bpm)	64.40±6.30	65.20±7.48	-0.230	0.822
SCR (mmol/L)	60.40±11.04	58.88±8.28	0.301	0.768
BUN (mmol/L)	5.38±1.32	4.88±1.17	0.750	0.466
TG (mmol/L)	1.20±0.23	1.12±0.52	0.312	0.760
TC (mmol/L)	4.42±0.56	4.76±0.80	-0.844	0.414
HDL (mmol/L)	1.40±0.35	1.55±0.45	-0.681	0.508
LDL (mmol/L)	3.04±0.65	3.14±0.75	-0.249	0.807
ALT (U/L)	20.80±11.20	20.00±13.94	0.117	0.908
AST (U/L)	23.40±13.85	23.60±13.07	-0.033	0.974
HbA1C (%)	5.57±0.27	5.54±0.27	0.215	0.833
FBG (mmol/L)	5.26±0.82	5.18±0.87	0.161	0.875
2h-BG (mmol/L)	6.73±1.79	6.60±2.06	0.122	0.904
FT3 (pmol/L)	4.28±0.49	4.16±0.51	0.420	0.682
FT4 (pmol/L)	14.34±1.62	13.66±1.47	0.822	0.426
TSH (mIU/L)	2.08±0.56	2.02±0.50	0.190	0.852
BUA (mmol/L)	226.40±54.99	243.80±27.70	-0.831	0.421
NT-proBNP (pmol/L)	755.40±578.01	52.40±19.83	3.998	0.002

MyBPC3-P459fs HCMs, hypertrophic cardiomyopathic patients carrying MyBPC3 P459fs mutation; HCs, healthy controls; BMI, body mass index; BSA, body surface area; SBP, systolic blood pressure; DBP, diastolic blood pressure; HR, heart rate; SCR, serum creatinine; BUN, blood urea nitrogen; Triglyceride, triglyceride; TC, total cholesterol; HDL, high density lipoprotein cholesterol; LDL, low density lipoprotein cholesterol; ALT, alanine transaminase; AST, aspartate aminotransferase; HbA1C, glycosylated hemoglobin A1c; FBG, fasting blood glucose; 2h-BG, 2h blood glucose; FT3, free triiodothyronine; FT4, free thyroxine; TSH, thyroid stimulating hormone; BUA, blood uric acid; NT-proBNP, N-terminal pro-brain natriuretic peptide.

Table S4 The differences of metabolites between P459fs cells and WT cells in metabolomic profiles.

Formula	Compounds	Class	VIP	*P* value	FC	Log2FC	Type
C12H22O11	Lactose	Sugars	1.344	0.001	2.798	1.484	↑
C12H22O11	Lactulose	Sugars	1.327	<0.001	2.656	1.409	↑
C6H12O4	Mevalonate	Organic acid and its derivatives	1.405	<0.001	4.080	2.023	↑
C45H90N2O6P+	SM(d16:1/24:1(15Z))	Sphingomyelins	1.406	<0.001	2.273	1.184	↑
C46H91N2O6P	SM(d17:1/24:1(15Z))	Sphingomyelins	1.398	0.002	2.659	1.411	↑
C43H87N2O6P	SM(d18:1/20:0)	Sphingomyelins	1.365	0.005	2.520	1.333	↑
C47H93N2O6P	SM(d18:1/24:1(15Z))	Sphingomyelins	1.408	<0.001	3.086	1.626	↑
C16H27NO14	L-Aspartic acid-O-diglucoside	Small peptides	1.259	0.004	2.028	1.020	↑
C42H81NO3	Cer(d18:1/24:1(15Z))	Ceramides	1.163	0.019	3.433	1.779	↑
C11H19NO9	N-Acetylneuraminic Acid(SA)	Amino acid derivatives	1.398	<0.001	0.460	-1.120	↓
C20H28O2	Vitamin A acid	Coenzyme and vitamins	1.361	0.001	0.246	-2.022	↓
C7H6O3	4-Hydroxybenzoic Acid	Phenolic acids	1.322	<0.001	0.358	-1.482	↓
C10H12O5	3,4,5-Trimethoxybenzoic Acid	Phenolic acids	1.302	0.002	0.477	-1.069	↓
C10H15N2O8P	Thymidine-5'-phosphate(dTMP)	Nucleotide and its metabolomics	1.387	<0.001	0.486	-1.040	↓
C10H14N5O6P	2'-Deoxyadenosine-5'-Monophosphate	Nucleotide and its metabolomics	1.394	0.004	0.228	-2.136	↓
C9H13N3O5	Cytidine	Nucleotide and its metabolomics	1.410	<0.001	0.408	-1.295	↓
C9H14N3O8P	Cytidine-5-Monophosphate	Nucleotide and its metabolomics	1.401	<0.001	0.489	-1.033	↓
C8H15NO3	N-Acetyl-L-Leucine	Amino acid derivatives	1.354	<0.001	0.481	-1.057	↓
C12H16N2O3	Ala-Phe	Small peptides	1.386	<0.001	0.384	-1.380	↓
C14H20N2O4	Val-Tyr	Small peptides	1.337	<0.001	0.341	-1.551	↓
C15H22N2O3	Leu-Phe	Small peptides	1.396	<0.001	0.406	-1.302	↓
C6H6N4O3	1-Methyluric Acid	Amino acid derivatives	1.395	<0.001	0.428	-1.224	↓
C22H32O3	(±)4-HDHA	Oxidized lipids	1.200	0.015	0.461	-1.118	↓
C20H32O3	(±)5-HETE	Oxidized lipids	1.388	<0.001	0.487	-1.038	↓
C20H32O3	(±)9-HETE	Oxidized lipids	1.388	<0.001	0.487	-1.038	↓
C15H28O2	FFA(15:1)	Free fatty acids	1.409	<0.001	0.125	-2.995	↓
C27H44NO7P	LPE(0:0/22:6)	Lysophosphatidylethanolamines	1.406	<0.001	0.461	-1.118	↓
C27H44NO7P	LPE(22:6/0:0)	Lysophosphatidylethanolamines	1.406	<0.001	0.461	-1.118	↓
C10H13N5O4	3-Deoxyguanosine	Nucleotide and its metabolomics	1.397	<0.001	0.479	-1.063	↓
C21H41O7P	LPA(18:1/0:0)	Lysophosphatidic acids	1.311	0.003	0.475	-1.073	↓
C11H22N2O3	Leu-Val	Small peptides	1.333	<0.001	0.454	-1.138	↓
C13H17N3O4	Phe-Asn	Small peptides	1.224	0.004	0.476	-1.072	↓
C26H46NO7P	LPC(18:4/0:0)	Lysophosphatidylcholines	1.265	0.028	0.467	-1.097	↓
C12H25N3O3	Ile-Lys	Small peptides	1.242	0.002	0.428	-1.225	↓
C10H13N4O7P	2'-Deoxyinosine-5'-monophosphate	Nucleotide and its metabolomics	1.407	<0.001	0.396	-1.336	↓

P459fs cells, H9C2 cells with MyBPC3 P459fs mutation; WT cells, H9C2 cells with MyBPC3 wild type; VIP, variable importance in the projection; FC, Fold change.

Table S5 The differences of proteins between P459fs cells and WT cells in proteomic profiles.

Accession	Gene symbol	*P* value	FDR	Sum peptide score	Coverage [%]	Unique peptides	Type
P23785	Grn	<0.001	High	86.164	39	1	↑
B2RZ78	Vps29	<0.001	High	39.966	65	1	↑
Q5XIE8	Itm2b	<0.001	High	29.518	35	6	↑
G3V8P5	RGD1310127	<0.001	High	12.934	11	3	↑
Q53B90	Rab43	<0.001	High	12.712	29	3	↑
Q62881	Nol3	<0.001	High	9.582	12	2	↑
D3ZM97	Olr1121	0.002	High	3.367	4	2	↑
A0A0G2K7P6		0.004	High	2.866	10	1	↑
P01946	Hba-a2; Hba1; Hba2	<0.001	High	6.85	13	1	↑
Q9QX67	Dap	0.002	High	3.241	8	1	↑
Q6P691	C2cd2	0.002	High	3.124	2	1	↑
F1LPQ4		0.012	Medium	1.993	1	1	↑
G3V6D8		<0.001	High	54.591	9	3	↓
Q566E5	Kdelc2	<0.001	High	38.884	21	11	↓
M0R4E1	Myl4	<0.001	High	32.896	44	6	↓
A0A0G2QC02	Skiv2l	<0.001	High	25.229	10	8	↓
B1PRL5	Murc; Cavin4	<0.001	High	22.947	23	7	↓
D3ZZ68	Synpo2l	<0.001	High	22.4	8	5	↓
Q63769	Srpx	<0.001	High	18.539	14	6	↓
Q5XIJ6	Babam1	<0.001	High	14.31	12	4	↓
Q5BK81	Ptgr2	<0.001	High	12.659	16	5	↓
B2RZ08	RGD1563325; Tmem263	<0.001	High	10.958	19	2	↓
P97531	Trip10	<0.001	High	7.778	5	3	↓
D3ZVN7	Ppox	<0.001	High	5.988	5	2	↓
D4A634		<0.001	High	11.155	3	2	↓

P459fs cells, H9C2 cells with MyBPC3 P459fs mutation; WT cells, H9C2 cells with MyBPC3 wild type; FDR, false discovery rate.
